# Inhalation of Ether in Instrumental Labor

**Published:** 1847-07

**Authors:** W. Channing


*A Case of Inhalation of Ether in Instrumental Labor.* By W. Channing, M. D.—I beg leave to offer you the following case for publication. It is indeed but a single instance of the use of ether in midwifery practice; still, such is the importance of that discovery which has abolished pain in so many, and in such a variety of cases—and such the state of opinion, and such the popular and professional interest, in everything bearing usefully on the subject, that I venture to present it in an amount of detail which otherwise might seem unnecessary. To my mind, in the present position of this great discovery this is the most proper method of communicating such facts.

I look back on the occurrences of this trial of ether with entire satisfaction, and with the deepest pleasure. The ether did just what was looked for from its use. It did it at once, and with no circumstances of embarrassment or difficulty. When its influence was no longer needed, its effects passed quietly away, and left a repose—a continued sense of relief, which, in an equal degree, and like kind, I do not remember to have witnessed before. I shall with pleasure communicate through your Journal the results of such farther trials of ether as circumstances may seem to authorize me to make. And no one should venture upon such trials until he is perfectly satisfied that such circumstances exist. A case came under my observation this day, which impressed upon my mind very strongly the importance of this rule of practice. It was one of unusual severity, and the time of suffering was long. Still there were circumstances in the previous history of my patient, and in her actual condition, which deterred me from taking ether with me. Such, however, at length, was the urgency, I may say violence, of demand for relief, on any terms, and for the use of ether especially, that I sent for it. I felt that the moral conviction, always so powerful in labor that relief would be obtained from this agent, might revive hope, and give encouragement, where a most depressing despair existed, and that thus the labor might be naturally terminated. Whether my reasoning were correct or not, I can say, that almost immediately after the messenger was despatched, efficient uterine contractions came on, which speedily, and safely, accomplished delivery.

Mrs. H., aged 23, was taken in labor, for the first time, May 5th, at 12 o'clock at night. I saw her between 9 and 10, of the morning of the 7th, in consultation with her medical attendant, Dr. W. E. Townsend. His pupil, Mr. Jerome Dwelley, was present, and who also from the beginning had faithfully attended to the case. The pains had been frequent and very severe. Some diminution of suffering had followed the exhibition of an opiate, which had been given before I saw the patient. Patient was well purged with castor oil day before labor. I found, on examination, the head fairly in the pelvis, where, I was told, it had been many hours. There was no *show*. The vagina was swollen, rough, hot, especially about the urethra, or anterior part of the pelvis. The os uteri was somewhat dilated, but less in its anterior portion than elsewhere, though in no part of its circumference had it cleared the head. It was swollen, smooth, hard, undilatable. It gave just that feel which so strongly intimates that the labor will be protracted, and accompanied by much suffering. The scalp was much swollen, and protruded as a tumor of a conical shape through the firm ring formed by the undilated and undilatable os uteri.

Mrs. H. was comparatively easy, from the opiate apparently. Her pulse was natural. Her strength was not much exhausted. Her stomach bore food well. There was no cerebral trouble, and the bladder had been duly emptied by the catheter. Under these circumstances I suggested delay; and it was agreed to wait to observe the changes which might occur in the present rest, and on the recurrence of pains. I saw her again at noon. Belladonna ointment was recommended, as no important change had occurred in the state of the os uteri. I was called to see her at about 6, P. M., about forty-two hours since labor began. I learned, on reaching the address, that the ointment had been used, and a solution of tartarized antimony exhibited, and that some change had occurred in the os uteri, namely, that it was more dilatable. Her pulse was now 120 in the minute. It was less strong than at noon. She could speak only in a whisper, and with great difficulty even so. She complained of great distress and most earnestly entreated to be relieved of her terrible suffering. On examination I found the os uteri somewhat more dilatable, and it was agreed that the forceps should be used.

Dr. Townsend called on me to make the visit just related. I said to him, in my study, that this seemed a very fair case for the use of ether. He agreed with me in this opinion, and added that he had a quantity of pure ether at home, and a sponge of suitable size foı its inhalation, and that he would meet me at his patient's house. We soon met there, and I proceeded to apply the forceps. I selected Davis's solid forceps, because they are narrow, thin, and very easily introduced, and seemed less likely to injure the os uteri than a broader and a thicker instrument. The application was perfectly easy, and I made an extracting effort, which was attended with very severe pain. Mrs. H. soon became quiet, and I desired Dr. T. to apply the sponge, saturated with ether, to the mouth and nose. This he did, and in about a minute she was under the full influence of the ether. The first inspiration produced a slight cough, as if the larynx had been irritated. It was like the sound by which an effort to remove some irritating matter from the air-passages is commonly accompanied. The next noticeable effect, and which was quite an early one, was a sudden movement of the body, such as is made sometimes when one is falling asleep, and has consciousness enough to know this, and to rouse the will into sufficient action to prevent it. It was involuntary, still it did not convey the idea of being spasmodic, in any morbid understanding of the term. She was directed to open her eyes, to answer questions, &c., but gave not the least evidence of consciousness of anything said. I now proceeded to extract. The os uteri at once came down again, and much embarrassed the operation, so that I desired Mr. Dwelley to pass his fingers between the shoulders of the forceps and the symphysis pubis, and gently press the protruding os uteri upwards. He did so and thus removed that part from the chance of injury. The extraction was continued at intervals. Not the smallest complaint was made. The womb was roused to action, and strong expulsatory efforts were made. The head advanced, and everything promised well. But at length the head became again firmly fixed, and this to a degree which prevented its being moved by any such force as I believed it safe to employ. I removed the forceps. The effect of the ether passed off, but as soon as consciousness returned, most earnest demands were made for more. "Put it to my mouth—I shall faint—you must;" in short, all forms of entreaty were made use of to obtain the entire relief that the ether had produced. She had at first refused to employ it. The ether had been now used up, and a short delay took place while a further supply was sent for. I perforated the cranium, fixed the hook, and made some extracting effort. Again was complaint made of the suffering which was immediately produced by the traction. The repose had been entire since consciousness had returned. She thought she was delivered. Said that she had *sense*, knew that she was alive, after the sponge was put to her mouth, but that she had no *feeling* after, and knew not what had happened. She had passed the time in most entire freedom from all pain. She said that there had been light before her eyes, and buzzing in her ears, and that she had been in another world. The aphonia had entirely disappeared, and her voice was natural. The ether was again applied to the mouth and nose, and when it was ascertained that its full effects were present, extracting effort was made by the hook. Again did the womb act, and the head advanced. Its progress was very slow. Much effort was demanded to bring the head along. The ether was used several times before the labor was over. In fact, she was most of the time inspiring the vapor, largely mixed with atmospheric air, for her pillow and bed-clothes were necessarily kept wet with it, from the mode of using it. There was no accident, or the least untoward circumstance attending the delivery. There was no pain—no complaint—no resistance of the effort used for delivery. The limbs were perfectly flaccid, and it was necessary that they should be kept separate by an assistant, and the whole weight of the upper one was to be supported. She came to herself soon after the child was born, and again expressed her entire ignorance as to everything that had been done. The placenta was separated, and reached the outlet by the unaided efforts of the womb, and no hemorrhage followed. A swathe was applied to the abdomen, and the patient made comfortable in her bed. I left soon after, having ascertained that her pulse was as good as it had been for some hours, and that everything promised well. It was impossible to determine what injury, if any, so long-continued pressure of the head had produced. The bladder had been carefully attended to, and the least possible amount of examination, I was told, had been made during the whole attendance on the case. The child had been dead some hours.

May 8th, 9, A. M.—I learned that soon after I left, the womb expelled from its cavity a large mass of coagula, with a gush of liquid blood. Cold was immediately applied to the abdomen, and the flow ceased. It was not so great as to affect at all her strength, or her pulse. I learned that she had passed an excellent night, and had slept as tranquilly as if under the kindest influence of opium. Her pulse was 108, of good strength and volume—tongue moist, head clear, and her whole state perfectly comfortable. We were particularly struck with these facts, in the distinct recollection of the long-continued suffering which a short time before had been endured. She had passed no water. The catheter was introduced with great ease, but got clogged with blood in its passage, so as to draw very little, if any urine. Mrs. H. said soon after that she felt a strong inclination to pass water, and in making an effort to do so there was expelled from the vagina a firm coagulum, and immediately after the urine followed voluntarily, and with perfect relief. Directions were given that the greatest quiet should be preserved, and sleep encouraged. Liquid farinaceous diet was ordered.

9th, A. M., 9 o'clock.—Mrs. H. slept most of yesterday, and less well last night. That is, was awake, but comfortable first part of night, slept latter part. Pulse now 104. Skin natural. No pain in abdomen, and no tenderness on pressure. Urine natural. Somewhat thirsty. Tongue slightly dry. No appearance of milk.

10th, 10, A. M.—Patient very comfortable. Pulse 108. Skin warm. Breasts distended and painful. Abdomen soft. Two dejections from ℥ ij. ol. ric., and as much lemon juice. In all respects doing well.

*Remarks.*—The ether was applied by a sponge. It was very easily applied. The effect was produced very soon, in about a minute, say after about fourteen respirations, and when consciousness was returning, one or two respirations were enough to procure insensibility. The room, or the atmosphere about the patient, was saturated with ether. Was there not danger of explosion had a candle or lamp been brought into this atmosphere? I have heard of experiments which were designed to prove that this fear is groundless. I have not seen them, and should be unwilling to act in accordance with them. In the knowledge that equal parts of the vapor of ether and atmospheric air, produced a compound as explosive as hydrogen and oxygen, he who uses ether at night should be most cautious to keep a lighted candle or lamp at a distance from the patient. As our midwifery arrangements so frequently occur at night, this may sometimes be an inconvenience. We cannot examine the pulse or the countenance during the use of ether, which it is very desirable to do. But we had better lose such opportunity, than incur the least risk of the explosion of the gas.

Cases are reported of instrumental labor in a Paris hospital under the use of ether, which were fatal by the supervision of puerperal fever. But this result will hardly be ascribed to the ether used, or be made an objection to its use elsewhere, as puerperal fever existed at the time in the hospital, and every body who knows anything of the disease, must be aware how readily it extends itself from patient to patient, especially in hospitals. It is said that this is especially true of the hospitals in Paris. I have not in memory a case of instrumental labor of so much severity as this above reported, from which recovery was so rapid, or so complete, and in which suffering was so slight. I do not recollect that a complaint was made of any suffering, from the time of the inhalation to the day on which I made my last visit.

Not only in Paris, but in Edinburgh also, has this method been tried in labor. To no one is the profession more indebted than to Dr. Simpson, Professor in the Edinburgh University, on this behalf. I quote from Forbes's Medical Review, the latest No., the leading authority in medical literature in Europe, the following on the subject. I do it for the facts to which it refers, and especially for the caution with which the information is accompanied. From the same Review I make an extract which represents the opinion of Dubois, with an important remark from the reviewer.

"In a communication which we have received from Edinburgh, dated the 22d of March, Dr. Simpson states that he had, up to that date, used etherization some forty or fifty times, with the most perfect safety and success. We understand that he has kept it up *for hours*—in one woman four, in another six hours—without the fœtal heart varying above ten or twelve beats during the whole time, the mother in both cases recovering perfectly, and both, of course, astonished at being delivered without being aware of it. We believe that Dr. Simpson, in making these statements, still inculcates caution in the use of the new means; justly regarding all his own trials hitherto, bold as they are, as merely experimental, and as only first fruits which, however delightful and promising, may not be the positive harbingers of an abundant and wholesome harvest.

"M. Dubois's opinion is, on the whole, not in favor of the employment of ether in midwifery, although he admits that he has seen no ill effects that he could, with certainty, attribute to it. He thinks, 'that it should be restrained to a very limited number of cases, the nature of which ulterior experience will better allow us to determine.' He, however, confesses that the result of the cases he has treated in this manner, has lessened the fears with which he originally entered on the trial. We leave the Professor and the Baron—the doughty champions and learned representatives of the obstetrics of Paris and Edinburgh—to fight the battle between them. Time, at least, will ere long determine which of the two is in the right. We are disposed to believe that neither is absolutely so; and that here, as in many other instances of clashing opinions, the truth lies between."

The action of the womb in the above case, in the absence of all voluntary agency, was very striking. Not only was there natural expulsatory effort, which was aiding the manual, but the effort was marked occasionally by its usual audible expression, the *bearing down* which is so well known. I was reminded of this effort during insensibility, by a case of most severe puerperal convulsions, which came under my notice the day after the above case. The organic effort, in the entire abolition of voluntary power, was most striking. I have known the child born by this organic agency, without the least apparent consciousness of the event on the part of the mother at the time, or memory of it afterwards. In this fact, established by so many, and so varied observations at home and abroad—in this fact of efficient uterine action produced by a well-known agent, ether, and the use of which has thus far been so safe, and the application and *modus operandi* of which, a wider observation will do more and more to determine—may we not in these facts look with confidence to the time when labor will be accomplished with an ease, a freedom from suffering, quite as great as has hitherto been the pain which has accompanied it, and which has been regarded as its necessary condition?—*Boston Med. and Surg. Journal.*

